# Physcion Induces Potential Anticancer Effects in Cervical Cancer Cells

**DOI:** 10.3390/cells10082029

**Published:** 2021-08-08

**Authors:** Wojciech Trybus, Teodora Król, Ewa Trybus, Anna Stachurska

**Affiliations:** 1 Laboratory of Medical Biology, Institute of Biology, The Jan Kochanowski University, Uniwersytecka 7, 25-406 Kielce, Poland; ewa.trybus@ujk.edu.pl; 2 Department of Immunohematology, Centre of Postgraduate Medical Education, Marymoncka 99/103, 01-813 Warsaw, Poland; anna.stachurska1@wp.pl

**Keywords:** physcion, apoptosis, mitochondria, autophagy, oxidative stress

## Abstract

Background: The extent of morphological and ultrastructural changes in HeLa cells was assessed by optical, fluorescence and electron microscopy after exposure to various concentrations of physcion, taking into account the biological properties of the test compound. Methods: Cell viability was assessed by MTT assay, while the cell cycle, LC3 expression, apoptosis, change of mitochondrial potential, Bcl-2 protein expression level and the level of reactive oxygen species were analyzed by flow cytometry. Results: As a result of physcion encumbrance, concentration-dependent inhibition of HeLa cell viability and the G0/G1 phase of the cell cycle was observed. Activation of the lysosomal system was also revealed, which was expressed by an increased number of lysosomes, autophage vacuoles and increased expression of the LC3 protein, a marker of the autophagy process. Transmission electron microscopy and fluorescence microscopy showed that physcion induced clear changes in cervical cancer cells, especially in the structure of the nucleus and mitochondria, which correlated with the production of reactive oxygen species by the test compound and indicated the induction of the oxidative process. At the same time, the pro-apoptotic effect of physcion was demonstrated, and this mechanism was dependent on the activation of caspases 3/7 and the reduction in Bcl-2 protein expression. Conclusion: The obtained results indicate an antitumor mechanism of action of physcion, based on the induction of oxidative stress, autophagy and apoptosis.

## 1. Introduction

Autophagy is the process of cellular component degradation taking place with the participation of the lysosomal system. On the one hand, this process may affect the survival of cancer cells, and on the other hand, it may promote cell death [1]. 

This dual role of autophagy can be used in the treatment of cancer. Numerous data indicate that autophagy is induced by various plant-derived chemotherapeutic agents, such as vinblastine [2], vincristine [3] and taxol [4]. Activating autophagy in cancer cells may be important in blocking the pathways leading to apoptosis, a process that is crucial in the elimination of cancer cells [5,6]. 

Other drugs used in anti-cancer therapy, such as 2-methoxyestradiol, doxorubicin or cisplatin, may increase oxidative stress and reduce the activity of antioxidant enzymes in cells, which may constitute an additional mechanism of tumor cell elimination [7].

Anthraquinones [8,9,10], including physcion, which is the subject of our research, may modify autophagy, apoptosis and induce oxidative changes in cancer cells.

Physcion occurs, i.a., in the roots of Rheum, and due to its antimicrobial [11], hepatoprotective [12], anti-inflammatory [13] properties is often used in traditional Chinese medicine [14]. 

Recent reports indicate that physcion, aloe-emodin, chrysophanol, rhein, danthron, hypericin and anthraquinone derivatives (daunorubicin, doxorubicin, epirubicin and diacerein) are potent inhibitors of SARS-CoV-2, including the main protease Mpro, papain-like protease ( PLpro) and RNA-dependent RNA polymerase (RdRp) [15].

Physcion also shows a potential antitumor effect, and its mechanism of action is most likely based on the induction of apoptosis [16] or inhibition of metastasis [17]. Such action has been demonstrated, among others, in hepatocellular carcinoma cells (HCC) [18], nasopharynx cancer (NPC) [16], or breast cancer [19]. 

The literature lacks data on the influence of physcion on ultrastructural and biochemical changes in cervical cancer cells, especially with regard to the lysosomal system and the related autophagy process, considered in the context of cell death as a result of autophagy or death accompanying autophagy.

## 2. Materials and Methods

### 2.1. In Vitro Culture Conditions 

The HeLa human cervical adenocarcinoma cells were purchased from the American Type Tissue Culture Collection (Rockville, MD, USA). HeLa cells were cultured at 37 °C and 5% CO_2_ atmosphere in a DirectHeat incubator (Thermo Fisher Scientific, Waltham, MA, USA) on a modified Dulbecco medium (GIBCO, New York, USA) containing 100 μg/mL penicillin-streptomycin (Corning, Manassas, USA), 10% fetal calf serum (Biowest, Nuaillé, France). Physcion (≥ 98.0%, TLC), 1,8-dihydroxy-3-methoxy-6-methylanthraquinone, was purchased from Sigma-Aldrich (St. Louis, MO, USA).

### 2.2. Assessment of Cell Viability 

The level of cytotoxicity of physcion in a concentration range of 80–300 (µM) on HeLa cells was measured using the MTT (3-(4,5-dimethyl-2-yl)-2,5-diphenyl-tetrazolium bromide) test. Cells with a density of 1 × 10^5^/mL grown in medium in a volume of 200 µM cultured in 96-well plates Falcon (Fisher Scientific, Waltham, MA, USA) were treated 24 h after passage with various concentrations of physcion 80–300 (µM). After 48 h of incubation, the cells were washed with PBS (Corning, Manassas, USA) and then MTT solution (Sigma Aldrich, St. Louis, MO, USA) was added to each well at a volume of 100 µL. After another 2 h of incubation, the MTT solution was filtered off, each well was rinsed with PBS, and then 100 µL of dimethyl sulfoxide (DMSO) (Sigma Aldrich, St. Louis, MO, USA) was added to solubilize the formazan crystals. Optical density was measured at 570 nm on a Synergy 2 microplate reader (BioTek, Winooski, VT, USA). The relative cell viability was calculated compared to the control group. The experiment was repeated three times.

### 2.3. Detection of Apoptosis Using Annexin V and Propidium Iodide (PI) Test

Analysis of the level of apoptosis in cells induced by physcion was performed using the fluorescein isothiocyanate (FITC)-annexin V/PI apoptosis detection kit (BD Biosciences, Franklin Lakes, NJ, USA). Cells were treated with physcion at 80 µM, 160 µM, 200 µM and 300 µM for 48 h, then separated from the medium using 0.25% trypsin-EDTA (Corning, Manassas, USA), centrifuged and washed with PBS. Cells were then incubated with 5 µL FITC/annexin V and 5 µL PI for 15 min at room temperature in the dark. Fluorescence intensity was analyzed using a FACSCanto II flow cytometer using FACSDiva software (BD Biosciences, San Jose, CA, USA). The experiment was repeated three times.

### 2.4. Assessment of Bcl-2 Protein Phosphorylation 

Changes in Bcl-2 phosphorylation in HeLa cells treated for 48 h with physcion were assessed by flow cytometry using the Muse™ Bcl-2 Activation Dual Detection kit (Merck-Millipore, Guyancourt, France) according to the manufacturer’s instructions. The kit contains two direct conjugate antibodies, phospho-specific anti-phospho-Bcl-2 (Ser70)-Alexa Fluor^®^555 and a conjugated anti-Bcl-2-PECy5, an antibody to measure the total expression level of Bcl-2. Assessment of the degree of activation of the Bcl-2 pathway was performed by measuring the phosphorylation of Bcl-2 relative to the total expression of Bcl-2 in the cells tested.

### 2.5. Analysis of Changes in Mitochondrial Membrane Potential

After 48 h of exposure to physcion at a concentration of 80 μM, 160 μM and 200 μM, cells were fixed for 10 min in 4% paraformaldehyde (PFA) solution in PBS and then incubated for 30 min with rhodamine 123 (RH 123) (Sigma Aldrich, St. Louis, MO, USA) at a concentration of 5 µg/mL ethanol, which is a fluorochrome with the ability to bind to metabolically active mitochondria. The cells were then washed with PBS and analyzed under a Nikon A1R confocal microscope based on a Nikon Eclipse Ti inverted microscope (Nikon Instruments Inc., Melville, NY, USA). The change in fluorescence emission was analyzed using Nikon Nis Elements AR software (Nikon Instruments Inc., Melville, NY, USA).

### 2.6. Measurement of the Mitochondrial Membrane Potential (Δψm) 

The Δψm reduction was analyzed with the Muse Mitopotential Assay kit (Merck Millipore). Cells treated with physcion (80–300 µM) were suspended in the Muse MitoPotential working solution and then incubated at 37 °C for 20 min. After incubation, cells were stained with 7-AAD at room temperature for 5 min. The stained cell suspension was analyzed by flow cytometry. The results were obtained from three independent replications. 

### 2.7. Measurement of the Production of Reactive Oxygen Species (ROS) 

To quantify the amount of HeLa cells subjected to oxidative stress, they were exposed to physcion at the concentrations of 80 µM, 160 µM, 200 µM and 300 µM. After 48 h of exposure, cells were stained using the Muse Oxidative Stress Kit (Merck—Millipore, Guyancourt, France) according to the manufacturer’s protocol. The results were obtained from three independent replications. 

### 2.8. Test for Caspase 3/7 Activity 

The level of apoptosis was based on caspase 3/7 activation, the activity of which was measured using the Caspase 3/7 test kit (Merck—Millipore, Guyancourt, France). After 48 h of treatment of the cells with the physcion at concentrations of 80 µM, 160 µM, 200 µM and 300 µM, cells were harvested by trypsinization. Then, cells were stained according to the protocol. The quantification of caspase-active cells was performed using a Muse^®^ analyzer (Merck—Millipore, Guyancourt, France).

### 2.9. Transmission Electron Microscopy

Cells exposed for 48 h to physcion at concentrations of 80 µM, 160 µM, 200 µM and 300 µM were fixed with 3% glutaraldehyde in cacodyl buffer (sodium cacodylate/ hydrochloric acid) (Serva Electrophoresis GmbH, Germany). Secondary fixation was carried out in 2% OsO_4_ (SPI, West Chester, PA, USA). The samples were then dehydrated at increasing alcohol concentrations, then embedded in Epon 812 epoxy resin (Serva Electrophoresis GmbH, Germany) and polymerized. Ultrathin sections were cut on a Leica EM UC7 ultramicrotome (Leica Biosystems, Wetzlar, Germany) and contrasted with uranyl acetate and lead citrate (SPI, West Chester, PA, USA). Preparations were analyzed by transmission electron microscope Tecnai G2 Spirit (FEI Company, Hillsboro, OR, USA) using a Morada camera (Olympus, Soft Imagine Solutions, Münster).

### 2.10. Detection of LC3 Antibodies

A kit based on Muse Autophagy LC3 (Merck—Millipore, Guyancourt, France) antibodies was used to detect autophagy. To study the effect of physcion on the modulation of the autophagy process, HeLa cells grown in 96 well plates were exposed to 48 h of anthraquinone at the concentrations of 80 µM, 160 µM, 200 µM and 300 µM. After this time, cells were incubated for 4 h with Autophagy Reagent A in EBSS medium (Corning). The cells were then washed with HBSS (Corning) and trypsinized. Cells were centrifuged, the supernatant was removed, and anti-LC3 Alexa Fluor^®^ 555 and Autophagy Reagent B were added to the cells. The samples were then incubated on ice for 30 min in the dark, then centrifuged and the supernatant removed. Cells were suspended in assay buffer and determined by flow cytometry. Cells that were treated with serum-free medium for 4 h were used as a positive control.

### 2.11. DAPI Staining 

Labeling of cell nuclei was performed using 4’,6-diamidino-2-phenylindole (DAPI). After 48 h of incubation of the cells with physcion at a concentration of 80 µM, 160 µM, 200 µM and 300 µM, 10 µg/mL DAPI (Sigma Aldrich, St. Louis, MO, USA) staining was performed. Preparations were analyzed using a Nikon Eclipse Ti fluorescence microscope (Nikon Instruments Inc., Melville, NY, USA) using a DAPI dichroic filter block (358 nm excitation, emission above 461 nm).

### 2.12. Cell Cycle Analysis

Cells were incubated for 48 h with physcion at 80 µM, 160 µM and 200 µM concentrations. To analyze DNA content, cells were fixed in 70% ethanol at −20 °C overnight, then suspended in PBS containing 40 μg/mL PI, 0.1 mg/mL RNase A and 0.1% Triton X-100 (Sigma Aldrich, St. Louis, MO, USA) in the dark for 30 min at 37 °C and analyzed using a FACSCanto II cytometer and the FACSDiva software (BD Biosciences, San Jose, CA, USA). The percentage of cells in each phase of the cycle was determined using ModFit LT 4.1.7 (Verity Software House, USA). The experiment was repeated three times.

### 2.13. Evaluation of Morphological Changes and Determination of the Mitotic Index

Cells (control and test) were grown on sterile coverslips in Falcon dishes (Fischer Scientific, Waltham, MA, USA). After a 48h exposure to physcion at concentrations of 80 µM, 160 µM, 200 µM and 300 µM, the cells were fixed in methanol and stained with Harris hematoxylin and eosin (Sigma Aldrich, St. Louis, MO, USA). Morphological analysis was performed using a Nikon Eclipse 80i microscope with Nikon NIS Elements D 3.10 software (Nikon Instruments Inc., Melville, NY, USA). In the preparations, 5000 cells were analyzed in three independent experiments (15,000 cells/concentration), of which the mean values were determined. The mitotic index was determined by counting dividing cells, expressed as a percentage.

### 2.14. Statistical Analysis 

Statistical data analysis was performed using one-way analysis of variance (ANOVA), with post hoc multiple comparisons using the Tukey test. *p* < 0.05 was considered statistically significant. Statistica 10.0 software (StatSoft, Krakow, Polska) was used for the data analysis.

## 3. Results

### 3.1. Induction of Apoptosis in HeLa Cells

**Figure 1:**
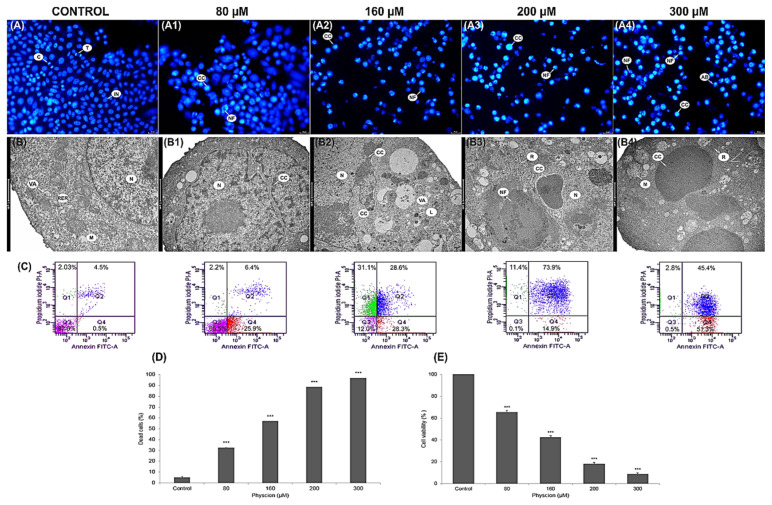
Physcion-induced apoptotic death of HeLa cells. Apoptosis observed in HeLa cells after physcion treatment. Labeling of cell nuclei with 4’,6-diamidin-2-phenylindole. (**A**) Control cells (unloaded) with normal nuclear morphology. Cells after 48 h of exposure to physcion at a concentration of 80 μM (**A1**), 160 μM (**A2**), 200 μM (**A3**) and 300 μM (**A4**) with visible chromatin condensation (CC), nuclear fragmentation (NF) and formation of apoptotic bodies (AB). T—Telophase, C—cytokinesis. Magnification, ×2000. Ultrastructural changes in cells treated with physcion at the concentrations of 80 μM, 160 μM, 200 μM and 300 µM. (**B**) Control cell with normal structure of the cell nucleus (N), with mitochondria with normal distribution of cristae (M), rough endoplasmic reticulum (RER) and small autophagic vacuoles (VA). Cells: with an altered nucleus shape (**B1**—80 µM), with visible condensation and marginalization of chromatin (**B2**—160 µM) and at a late stage of apoptosis (**B3**—200 µM, **B4**—300 µM) with condensed cytoplasm, condensed and fragmented chromatin (CC) and with a fragmented nucleus (NF). M—swollen mitochondria, R—free ribosomes, L—lysosomes. Magnification ×11,500. (**C**) Apoptosis assessed by annexin V-FITC/PI staining. The cells were treated for 48 h with physcion at the concentrations of 80 µM, 160 µM, 200 µM and 300 µM. Control cells (not in apoptosis) without Annexin V-FITC and PI staining. Populations of cells after physcion treatment: live cells (annexin V-FITC-/PI-), cells in the early phase (annexin V-FITC+/PI-) and late phase apoptosis (annexin V-FITC+/PI+), dead cells (Annexin V-FITC/PI+). (**D**) Percentage of early and late apoptotic cells induced by physcion in the concentration range of 80 µM–300 µM. (**E**) Cell viability measured by MTT after exposure to physcion in concentrations of 1-300 µM. Representative data from three parallel experiments. Data correspond to mean values ± standard error (S.E.). The differences were statistically confirmed at the level: *** *p* < 0.001.

Morphological analysis of the nuclei of DAPI stained cells (Figure 1(A1–A4)) showed a significant increase in the number of apoptotic cells at all physcion concentrations (80 µM, 160 µM, 200 µM and 300 µM). In the photos assessed (Figure 1(A1–A4)), condensed chromatin was visible, with a clear fragmentation of the nuclei with numerous apoptotic bodies. The greatest range of changes was found at the concentration of 300 µM (Figure 1(A4)). Ultrastructural analysis of the studied cells also revealed changes characteristic of programmed cell death, such as: change in the shape of the nucleus (Figure 1(B1)), condensation and marginalization of chromatin (Figure 1(B2)), cell shrinkage and nuclear fragmentation (Figure 1(B3–B4)).

The cytometric analysis showed that the encumbranced of physcion cells at the concentration of 80 µM and 160 µM increased the number of apoptotic cells to 32.5% (*p* ≤ 0.0001) and 56.9% (*p* ≤ 0.0001) in relation to the number of apoptotic cells in the control group (5.1%). Physcion in a concentration of 200 µM enhanced apoptotic processes; the number of apoptotic cells increased to 88.5% (*p* ≤ 0.0001). On the other hand, at the concentration of 300 μM, the highest increase in the number of apoptotic cells to 96.73% (*p* ≤ 0.0001) was demonstrated, mainly with the late apoptosis phenotype (Annexin V-FITC+/PI+) (Figure 1C,D).

### 3.2. Inhibition of the Viability of HeLa Cells through Mitochondrial Dysfunction

Physcion reduced the survival rate of cervical cancer cells in a concentration-dependent manner (Figure 1E). The values showing the inhibition of cell viability were statistically significant at the concentration of 80 μM—65.41%, 160 μM—42,33% and 200 µM—18.03%, respectively. The highest decrease in cell viability by over 90% compared to the control was demonstrated at the concentration of 300 µM; live cells constituted only 8.6%.

At a lower concentration (80 µM) of the test compound, a reduction in mitochondrial function was observed, which was expressed as a reduced reduction of MTT dye to formazan. On the other hand, the changes revealed at physcion levels of 160–300 µM indicated a significant damage to the mitochondria (Figure 1) and could have had an effect on the increased rate of apoptosis.

### 3.3. Changes in Caspase 3/7 Activity

**Figure 2:**
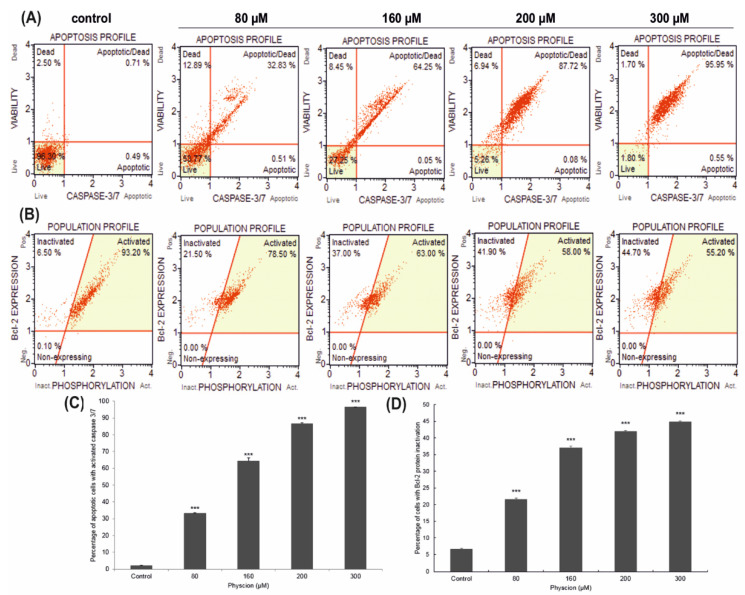
Effect of physcion on caspase 3/7 activity and Bcl-2 levels. Cells were treated for 48 h with physcion at concentrations of 80 µM, 160 µM, 200 µM and 300 µM (**A**). Live cells (caspase 3/7-/7-AAD-), early apoptotic cells (caspase 3/7+/7-AAD-), late apoptotic (caspase 3/7+/7-AAD+), dead cells (caspase 3/7-/7-AAD+) (**B**). Bcl-2 expression profile in physcion-treated HeLa cells. Cells expressing Bcl-2 are clustered in the top two quadrants of the scatterplot (inactive and activated). As a result of the action of physcion in increasing concentrations, an increased number of cells with inactivation of the Bcl-2 protein was observed. At a concentration of 300 µM, more than 44% were dephosphorylated, which confirms the inactivation of the Bcl-2 signaling pathway. Concentration-dependent percentage of cells with Bcl-2 protein inactivation (**C**) and apoptotic cells with activated caspase 3/7 (**D**). Data representative of three parallel experiments correspond to mean values ± standard error. The differences were statistically confirmed at: *** *p* < 0.001.

At a concentration of 80 µM and 160 µM, 33.06% (*p* ≤ 0.0001) and 64.38% (*p* ≤ 0.0001) of apoptotic cells were shown, respectively. Increased induction of apoptosis was observed with the increased concentration of physcion. The highest activity of caspase 3/7 was found at the concentration of 300 µM, where over 96% of cells (*p* ≤ 0.0001) were cells with caspase activity. These results indicate a physcion concentration dependent, pro-apoptotic effect (Figure 2A).

### 3.4. Effect of Physcion on the Inactivation of Bcl-2

The action of physcion reduced the phosphorylation of Bcl-2 protein in HeLa cells, which determined the survival of cancer cells and was reflected in an increased number of cells with inactivation of the above-mentioned protein. At 80 µM of physcion concentration, more than 21% of cells showed inactivation of the Bcl-2 protein (Figure 2B). Statistically significant changes (*p* ≤ 0.0001) were found at increasing concentrations of 160, 200 and 300 μM, where cells with inactivated Bcl-2 protein accounted for 37%, 41.90% and 44.70%, respectively.

### 3.5. Progressive Mitochondrial Damage

**Figure 3:**
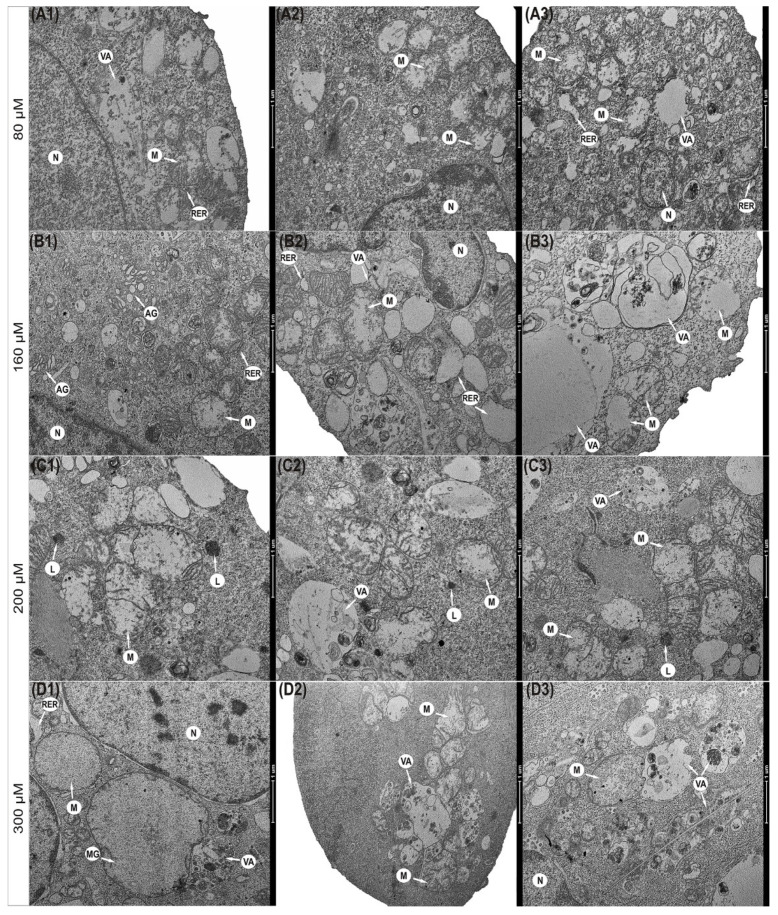
Ultrastructural changes in mitochondria of HeLa cells encumbered for 48 h with physcion. (**A1**–**A3**) Cell with swollen mitochondria and dilated channels of rough endoplasmic reticulum (80 µM). (**B1**–**B3**) Swollen mitochondria with clear matrix, short cristae (160 µM), mitochondria with membrane rupture and infused into the cytoplasm with 200 µM (**C1**–**C3**) and 300 µM (**D1**–**D3**). Megamitochondria present in the cytoplasm (**D1**) (300 µM) with complete loss of mitochondrial cristae. N—Nucleus, M—mitochondria, MG—megamitochondria, AG—Golgi apparatus, RER—rough endoplasmic reticulum, VA—autophagic vacuoles, L—lysosomes. Magnification ×11,500.

Physcion also caused significant changes in the morphological profile of mitochondria (Figure 3). It was found that mitochondria in the cells exposed to the 80 µM concentration were significantly enlarged with a slight brightening of the matrix (Figure 3(A1–A3)). The concentration of 160 µM anthraquinone caused progressive swelling of mitochondria. Mitochondria had a clear matrix and significantly reduced mitochondrial cristae (Figure 3(B1–B3)). In some mitochondria, interrupted membrane continuity was revealed (Figure 3(B3)). The consequence of the action of physcion at 200 μM (Figure 3(C1–C3)) and 300 μM (Figure 3(D1–D3)) was also a high-amplitude swelling of mitochondria and misplaced cristae. Some mitochondria showed a disruption of the membrane with concomitant leakage of contents into the cytoplasm (Figure 3(C2–C3)). Cells also contained megamitochondria, with a translucent matrix and virtually invisible combs drawn into the swollen membrane (Figure 3(D1)). 

### 3.6. Change in the Membrane Potential (ΔΨm) of HeLa Mitochondria

**Figure 4:**
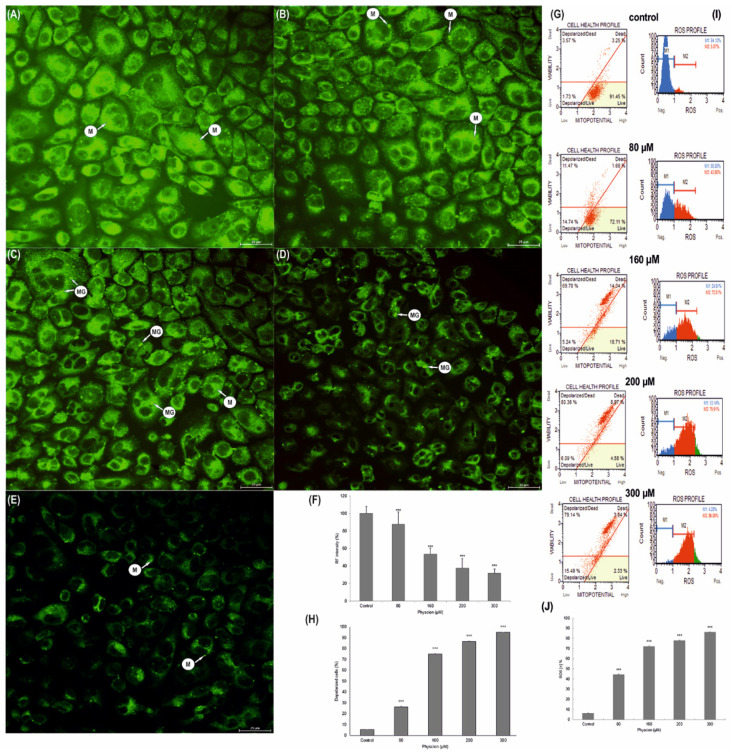
Morphological and biochemical changes in mitochondria of Hela cells after physcion treatment. Mitochondrial membrane potential change analysis based on rhodamine 123 staining. Representative photos of control cells with high mitochondrial membrane potential (**A**) and cells incubated for 48 h with physcion at concentrations of 80 µM (**B**), 160 µM (**C**), 200 µM (**D**) and 300 µM (**E**). Relative fluorescence intensity expressed as a percentage of the control group (**F**). Data correspond to mean values ± S.E. M—mitochondria, MG—megamitochondria. Magnification, ×4000. Changes in mitochondrial membrane potential dependent on physcion concentration (**G**). Percentage of cells with depolarization of the mitochondrial membrane (**H**). Each sample was analyzed in triplicate. The differences were statistically confirmed at: *** *p* < 0.001. Generation of reactive oxygen species (ROS) by physcion at concentrations of 80 µM, 160 µM, 200 µM and 300 µM (**I**). Percentage of ROS (+) cells induced with physcion (**J**). The degree of ROS production in cells was determined in comparison with the control. Each sample was analyzed in triplicate. The differences were statistically confirmed at: *** *p* < 0.001.

Correlated with the increase in anthraquinone concentration, the quenching of green fluorescence emission indicates depolarization of the mitochondrial membrane (Figure 4). This was manifested by a loss of pigment that had the ability to accumulate in the mitochondria. A significant disappearance of emissions (up to 37.55%, *p* ≤ 0.0001) was demonstrated at a concentration of 200 µM, and the highest at a concentration of 300 µM physcion (31.43%, *p* ≤ 0.0001) (Figure 4D–E). Normal control cell mitochondria with high membrane potential showed intense green fluorescence (Figure 4A). Physcion also caused the appearance of megamitochondria in the cytoplasm of the studied cells (Figure 4C–D).

The observed changes in rhodamine-labeled mitochondria correlated with a decrease in the potential of mitochondrial membranes, which followed the concentration of physcion, where the highest depolarization of mitochondrial membranes was demonstrated at a concentration of 200 µM (86.55%, *p* ≤ 0.0001), and 300 µM (over 94 %, *p* ≤ 0.0001) (Figure 4G,H).

### 3.7. Increase in Production of ROS in HeLa Cells

The estimation of the percentage of HeLa cells exposed to oxidative stress as a result of their 48-h exposure to physcion was based on the intracellular detection of superoxide radicals. In histograms showing the population of control and test cells stained with a specific ROS dye, ROS-producing cells are referred to as ROS (+), while cells that did not bind the dye are referred to as ROS (−). The research showed a significant (*p* ≤ 0.0001) increase in the production of ROS, generated by the action of physcion at all tested concentrations, compared to control cells (6.21%): up to 44.42% at a concentration of 80 µM, up to 71.98% at a concentration of 160 µM and up to 77.81% at a concentration of 200 µM. At the highest concentration used, 300 µM, the ROS (+) cell population increased to 86.05% (Figure 4G). The obtained results indicate a strong pro-oxidative effect of physcion on cervical cancer cells.

### 3.8. Induction of Autophagic Processes

**Figure 5:**
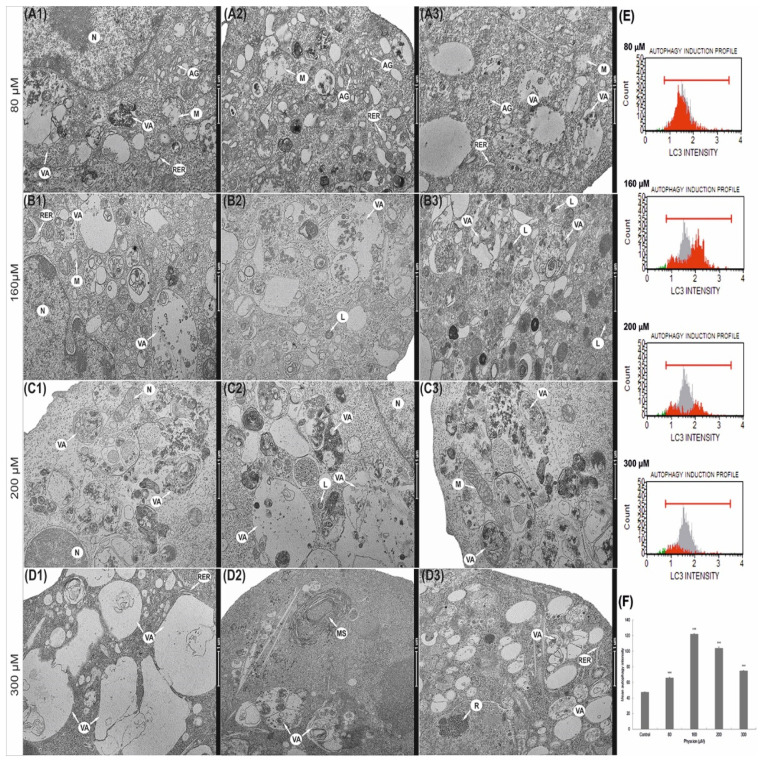
Ultrastructural and biochemical changes in the lysosomal compartment of HeLa cells encumbered with 48 h of physcion action. (**A1**–**A3**) Cells with numerous autophagic vacuoles and swollen cisterns of Golgi apparatus (80 µM). Cells with intense degradation processes, with numerous autophagic vacuoles and lysosomes (160 µM) (**B1**–**B3**). Vacuoles at various stages of digestion occupying the entire area of the cell 200 μM (**C1**–**C3**) and 300 μM (**D1**–**D3**). N—nucleus, M—mitochondria, MG—megamitochondria, AG—Golgi apparatus, RER—rough endoplasmic reticulum, VA—autophagic vacuoles, L—lysosomes. Magnification, ×11,500. Physcion increased the expression of the LC3 protein. (**E**) Histograms of HeLa cells treated for 48 h with physcion at concentrations of 80–300 µM. Cells were stained with conjugated anti-LC3/Alexa Fluor^®^555 antibody, and the fluorescence intensity was measured cytometrically. (**F**) Change of fluorescence intensity in HeLa cells with LC3 protein expression depending on physcion concentration. The results are the mean of three independent experiments. The differences were statistically confirmed at the level: *** *p* < 0.001.

The increasing concentration of physcion resulted in the intensification of autophagy, expressed in an increase in the number of primary and secondary lysosomes and numerous autophage vacuoles (Figure 5). Already at a concentration of 80 µM, an increase in the number of vacuoles in the cytoplasm was shown, as well as a slight swelling of the rough endoplasmic reticulum cisterns (Figure 5(A1–A3)). At a concentration of 160 μM, numerous vacuoles of various sizes and shapes were visible (Figure 5(B1–B3)). The vacuoles were filled with material, at various stages of digestion they contained fragments of cytoplasm and cell organelles. Progressive macroautophagy was observed at 200 µM (Figure 5(C1–C3)) and 300 µM (Figure 5(D1–D3)). In the cytoplasm of the studied cells, apart from numerous vacuoles, myelin structures were also observed (Figure 5(D2)). The photomicrographs showed swollen cisterns of the Golgi apparatus (Figure 5(A1–A2)) Noteworthy are the significantly widened cisterns and a rough endoplasmic reticulum, often devoid of ribosomes (Figure 5(A2,B1)).

### 3.9. Physcion Promotes Autophagy by Increasing the Level of LC3 Protein

Depending on the concentration, physcion promotes autophagy in HeLa cells, which is expressed, i.a., by increasing the level of the LC3 protein-a marker of autophagy.

At 48 h of treatment with physcion, HeLa cells significantly increased the number of autophage LC3-positive cells (Figure 5E–F). The mean values of the intensity of autophagy for the controls were 47.4 (gray area). However, after incubating HeLa cells with the test compound at a concentration of 80 µM, the mean fluorescence intensity (red area) increased to 65.5 and to 121.5 at a concentration of 160 µM (Figure 5). At 200 µM and 300 µM physcion concentrations, a slight decrease in fluorescence intensity was shown to 103.8 and 74.7, respectively, but these values were significantly higher compared to the control. Cytometric analysis showed that physcion-induced autophagy is the process that ultimately leads to apoptosis at its highest concentrations (200 and 300 µM), as also confirmed by caspase 3/7 activation shown in Figure 2A. 

### 3.10. Increased Vacuolization

**Figure 6:**
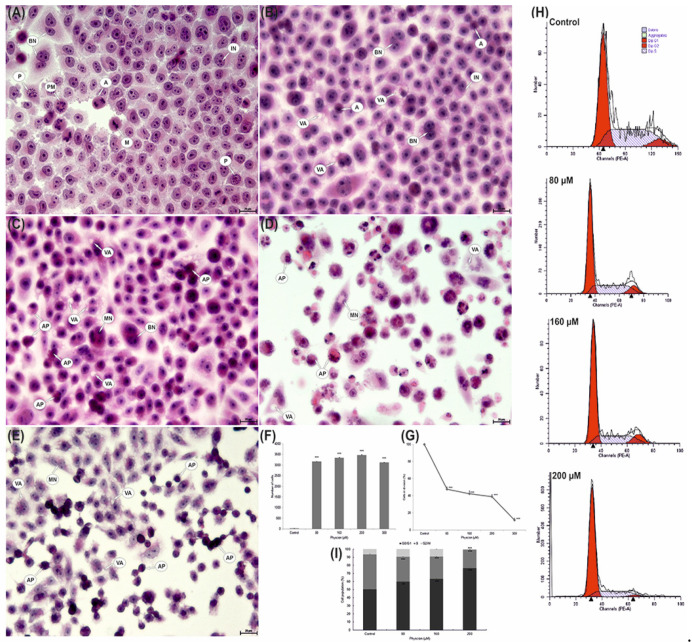
Morphological changes in HeLa cells after 48 h of exposure to physcion. (**A**) Control cells with normal morphology and numerous cells in division. (**B**) Cells with vacuoles present in the cytoplasm (80 µM). Numerous cells with strong cytoplasmic vacuolization covering the entire cell area and numerous apoptotic cells (**C**—160 µM, **D**—200 µM and **E**—300 µM). IN—interphase, P—prophase, PM—prometaphase, M—metaphase, A—anaphase, AP—apoptotic cells, VA—cells with cytoplasmic vacuolization, BN—binuclear cells, MN—multinucleated cells. Hematoxylin and eosin staining. Magnification, ×4000. (**F**) Number of cells with cytoplasm vacuolization. (**G**) Mitotic index of HeLa cells. Data correspond to mean values ± S.E. from three different experiments. The differences were confirmed statistically at: *** *p* < 0.001. (**H**) Effect of physcion at concentrations (80 µM, 160 µM and 200 µM) on cell cycle progression in HeLa cell lines. (**I**) Percentage of cells in different phases of the cell cycle. Cell cycle distribution was analyzed by flow cytometry. Data correspond to mean values ± S.E. from three different experiments. The differences were confirmed statistically at: *** *p* < 0.001.

Morphological analysis revealed a relationship between the concentration of physcion and the severity of vacuolisation in the cytoplasm of the studied cells. In the case of apoptosis, an increase in the concentration of physcion caused an increase in vacuolization (Figure 6B–E). At an 80 μM anthraquinone concentration, a statistically significant vacuolization (3164 cells; 63.28% of analyzed cells) was observed (*p* ≤ 0.0001). Progressive vacuolization changes were observed at concentrations of 160 μM and 200 μM, showing 3341 (66.82%; *p* ≤ 0.0001) and 3463 (69.29%; *p* ≤ 0.0001), respectively, vacuolated cells compared to control cells (0.34%). A slight decrease in their number was demonstrated at a concentration of 300 µM, which could be due to the increase in the number of cells in apoptosis (Figure 6E). It was also a statistically significant result (*p* ≤ 0.0001).

### 3.11. Reduction of the Mitotic Index of HeLa Cells

As a consequence of incubation of HeLa cells with physcion, a decrease in the value of the mitotic index was observed. At 80 μM physcion concentration, the value of the mitotic index was 47.48% (*p* ≤ 0.0001). A progressive decrease in cell viability was demonstrated at concentrations of 160 µM (42.35%) and 200 µM (39.04%). 

The lowest percentage of cells during division (11.64%) in relation to the mitotic index of control cells (assumed as 100%) was found at 300 μM concentration of the tested factor (Figure 6G).

### 3.12. Changes in Cell Distribution in the Cycle

Cell cycle analysis showed a physcion concentration-dependent increase in the number of cells accumulated in the G0/G1 phase. A statistically significant increase in cells up to 59.71% (*p* ≤ 0.0002) and up to 63.38% (*p* ≤ 0.0001) in the G0/G1 phase was demonstrated at the concentration of 80 μM and 160 μM compared to the control (50.33%) (Figure 6). An increased accumulation of cells was demonstrated in the G0/G1 phase at a concentration of 200 µM; they constituted over 76% (*p* ≤ 0.0001).

## 4. Discussion

Both the problem of induction of death and inhibition of cell survival are currently the main goals of anticancer therapy. Additionally, resistance to chemotherapeutic agents is a significant problem in oncology, as it may reduce the effectiveness of anticancer drugs [20]. 

Therefore, the question of how to improve oncological treatment to avoid cell resistance to cytostatics is still asked. At present, there is still no clearly defined role of autophagy in cancer therapy, so it is important to pay attention to this important process in the cell. Understanding its new function can lead to the development of a promising therapeutic strategy to increase the effects of chemotherapy and improve clinical results in the treatment of cancer patients [20]. 

Numerous experiences show that anticancer drugs, including rituximab [21], bortezomib [22], cyclosporine [23] or rapamycin [24], often induce autophagy, especially in cells with impaired apoptosis and excessively damaged cells that try to counteract damage through progressive autophagy, which may promote autophagic cell death [25]. 

Interestingly, we have also demonstrated the induction of autophagy as a potential anti-cancer mechanism in relation to other previously studied anthraquinones, obtained from aloe: aloe-emodin [8], emodin [10] and chrysophanol [26]. Autophagy in neoplastic cells was also observed as a result of the action of other compounds of plant origin, including curcumin [27,28], celastrol [29], berberine [30] or honokiol [31]. 

Since autophagy, depending on the factors acting on the cell, can play a double role, it can protect the cell or lead it to death; therefore, our research attempted to answer the question of whether and how physcion affects the autophagy process in cancer cells.

Numerous lysosomes and various autophage vacuoles were found in the ultrastructure of HeLa cells exposed to physcion, and their number and characteristic morphological differentiation indicated ongoing macroautophagy (Figure 5).

This process was confirmed by an increase in the expression of the LC3-II protein, a marker of autophagy; this protein creates a stable connection with the autophagosome double membrane and is responsible for its extension [32,33]. 

The revealed changes were also confirmed by optical microscopy, where at the highest physcion concentrations (200 and 300 µM), the cytoplasm of the tested cells was strongly vacuolated (Figure 6).

The characteristic submicroscopic changes in cervical cancer cells after physcion loading support the induction of autophagous death through its excessive stimulation, which may be an additional method of eliminating neoplastic cells.

Additionally, Baehrecke [34] in his work suggests that the excessive presence of autophagosomes in dying cells is an indicator of programmed type II death, which may indicate an important role of autophagy in cancer therapy.

The literature data [35] show that the increased induction of autophagy in neoplastic cells precedes apoptotic death. Additionally, in our experiment, using microscopic and biochemical techniques in neoplastic cells incubated with physcion (especially in the highest concentrations, i.e., 300 µM), the presence of cells with apoptotic features was demonstrated (Figure 1 and Figure 2).

On the other hand, the photomicrographs in Figure 6 D and E document the presence of strongly contracted cells with dense cytoplasm and highly condensed chromatin. The use of transmission electron microscopy enabled the identification of cells at various stages of apoptosis, both early and late (Figure 1(B1–B4)). The presence of cells characteristic of apoptosis was confirmed by DAPI staining which showed a large number of cells with chromatin condensation and fragmentation of the cell nucleus (Figure 1(A1–A4)). The obtained morphological changes correlated with biochemical changes, i.e., with an increase in the activity of caspases 3/7, the executive enzymes of the apoptotic process, and with the inactivation of the Bcl-2 protein (Figure 2).

At the same time, it should be emphasized that the induced apoptotic death in the studied cells is mediated by mitochondria, as indicated by a decrease in the metabolic activity of mitochondria (as shown in the MTT viability test, Figure 1E), a decrease in the potential of mitochondrial membranes and an increase in the permeability of mitochondrial membranes (Figure 4G), as well as a decrease in the intensity of mitochondria seen in the emission of rhodamine 123 (Figure 4C,D). Micrographs documenting ultrastructural changes in the studied cells showed that with an increasing physcion concentration, mitochondrial edema, damage to mitochondrial membranes and megamitochondria are present (Figure 4). These changes correlated with an increase in reactive oxygen species, as shown in Figure 4I.

Cytostatics, including those of plant origin, used in oncological therapy often induce a cytotoxic effect on cancer cells through the production of free radicals, which, by contributing to the damage of membranes in various cell compartments, increase the level of oxidative stress, which, in consequence, may activate the process of apoptosis, or autophagy in cancer cells.

Contemporary oncological therapy focuses on anti-cancer strategies with the use of pro-oxidative compounds, the action of which accelerates the accumulation of ROS, disturbs redox homeostasis and causes serious damage to cancer cells [7]. 

In addition to its broad-spectrum activity on cervical cancer cells, physcion also has an inhibitory effect on cell cycle progression and cell inhibition in the G0/G1 phase (Figure 6H). The reduction in the mitotic index demonstrated in the studies (Figure 6G) demonstrates the concentration-dependent antiproliferative effect of physcion.

The multifaceted research results presented in our study have shown that physcion has a concentration-dependent effect on the processes of apoptosis and autophagy, induces oxidative stress and affects the cell cycle, and the possibility of modifying these processes important for cells may be of significant importance in anti-cancer therapy.

## 5. Conclusions

In summary, physcion has a cytotoxic effect on HeLa cells. The induction of apoptosis and autophagy demonstrated in studies may be a common mechanism for the antitumor effect of physcion on HeLa cells. The obtained results suggest that physcion in the future may find application in cancer therapy; however, this problem requires further research.

## Data Availability

The data that support the findings of this study are available from the corresponding author upon reasonable request.
